# Supplementary material for: Measuring the readiness to screen and manage intimate partner violence: Cross-cultural adaptation and psychometric evaluation of the PREMIS tool for perinatal care providers
Source: PLoS One. 2021 Nov 4;16(11):e0258943. doi: 10.1371/journal.pone.0258943 (PMC8568123; doi:10.1371/journal.pone.0258943)
Supplement: S1 Table — (DOCX) [file pone.0258943.s001.docx]

**S1 Table: Sociodemographic and professional characteristics of participants**

| Participants’ characteristics | Participants  N=360 | Test-retest participants  N=24 |
| --- | --- | --- |
| Age (years), *n (%)* |  |  |
| 20-29 | 32 (8.9) | 4 (16.7) |
| 30-39 | 112 (31.1) | 3 (12.5) |
| 40-49 | 97 (26.9) | 9 (37.5) |
| 50-59 | 93 (25.8) | 5 (20.8) |
| 60-69 | 26 (7.2) | 3 (12.5) |
| Women, *n (%)* | 337 (93.9) | 20 (83.3) |
| Field of practice, *n (%)* |  |  |
| Internal medicine | 1 (0.3) | 0 |
| General practitioner | 25 (6.9) | 0 |
| Pediatrics | 44 (12.2) | 1 (4.2) |
| Psychiatry | 4 (1.1) | 0 |
| Surgery | 1 (0.3) | 0 |
| Gynecology and obstetrics | 244 (67.8) | 20 (83.3) |
| Mother and child protection services | 29 (8.1) | 2 (8.3) |
| Other | 32 (8.9) | 1 (4.2) |
| Number of years practicing, *mean ± SD* | 17.9 *±* 10.8 | 18.9 ± 11.0 |
| Number of patients per week, *n (%)* |  |  |
| Not seeing patients | 14 (3.9) | 1 (4.3) |
| Less than 20 | 111 (31.0) | 7 (30.4) |
| 20-39 | 122 (34.1) | 9 (30.1) |
| 40-59 | 53 (14.8) | 2 (8.7) |
| 60 or more | 58 (16.2) | 4 (17.4) |
| Previous IPV training, *n (%)* |  |  |
| None | 85 (23.6) | 1 (4.2) |
| Institution’s protocol | 39 (10.8) | 5 (20.8) |
| Video | 99 (27.5) | 8 (33.3) |
| Lecture or talk | 162 (45.0) | 16 (66.7) |
| Skills-based training or workshop | 82 (22.8) | 9 (37.5) |
| School classroom setting | 66 (18.3) | 8 (33.3) |
| School clinical setting | 10 (2.8) | 0 |
| Continuing medical education program | 75 (20.8) | 5 (20.8) |
| Other in-depth training (more than 4 hours) | 64 (17.8) | 5 (20.8) |
| Other | 27 (7.5) | 4 (16.7) |
| If previous IPV training, total number of hours, *n (%)* |  |  |
| < 10h | 117 (50.0) | 10 (47.6) |
| 10h-19h | 56 (23.9) | 5 (23.8) |
| ≥ 20h | 61 (26.1) | 6 (27.3) |
| IPV experience in the last 6 months ^*^, *n (%)* |  |  |
| Yes | 205 (63.7) | 13 (59.1) |
| No | 117 (36.3) | 9 (40.9) |

^*^ At least one new diagnose of IPV (picked up an acute case, uncovered ongoing abuse, or had a patient disclose a past history) made in the last 6 months
