# Supplementary material for: Measuring the readiness to screen and manage intimate partner violence: Cross-cultural adaptation and psychometric evaluation of the PREMIS tool for perinatal care providers
Source: PLoS One. 2021 Nov 4;16(11):e0258943. doi: 10.1371/journal.pone.0258943 (PMC8568123; doi:10.1371/journal.pone.0258943)
Supplement: S2 Table — (DOCX) [file pone.0258943.s002.docx]

**S2 Table: Corrected item-total correlations for the PREMIS-French “Opinions” subscales**

| PREMIS-French “Opinions” subscales | Corrected item-total correlations | | | | | | |
| --- | --- | --- | --- | --- | --- | --- | --- |
|  | Item | Preparation | Workplace issues | Legal requirements | Self-efficacy | Alcohol/  drugs | Victim understanding |
| Preparation | 6 | **0.56** | 0.38 | 0.34 | 0.41 | -0.06 | 0.12 |
|  | 9 | **0.63** | 0.60 | 0.46 | 0.57 | 0.07 | 0.16 |
|  | 10a | **0.85** | 0.49 | 0.37 | 0.45 | 0.02 | 0.20 |
|  | 10b | **0.70** | 0.35 | 0.32 | 0.39 | -0.01 | 0.16 |
|  | 10c | **0.82** | 0.43 | 0.31 | 0.39 | -0.01 | 0.21 |
| Workplace issues | 2 | 038 | **0.57** | 0.24 | 0.34 | 0.10 | 0.12 |
|  | 3 | 0.44 | **0.61** | 0.37 | 0.33 | 0.03 | 0.16 |
|  | 4 | 0.41 | **0.48** | 0.38 | 0.30 | 0.09 | 0.13 |
|  | 18 | 0.32 | **0.49** | 0.22 | 0.30 | 0.18 | 0.15 |
|  | 19 | 0.42 | **0.48** | 0.37 | 0.45 | 0.07 | 0.03 |
|  | 25 | 0.33 | **0.44** | 0.34 | 0.38 | 0.10 | 0.04 |
| Legal requirements | 12a | 0.49 | 0.46 | **0.69** | 0.48 | 0.07 | 0.14 |
|  | 12b | 0.36 | 0.40 | **0.62** | 0.32 | 0.11 | 0.09 |
|  | 12c | 0.22 | 0.18 | **0.50** | 0.28 | 0.08 | -0.02 |
|  | 17 | 0.26 | 0.38 | **0.43** | 0.29 | 0.11 | 0.05 |
| Self-efficacy | 5 | 0.28 | 0.21 | 0.25 | **0.49** | 0.12 | 0.02 |
|  | 14 | 0.57 | 0.49 | 0.45 | **0.57** | 0.11 | 0.09 |
|  | 26 | 0.50 | 0.57 | 0.43 | **0.54** | 0.14 | 0.10 |
|  | 32 | 0.19 | 0.19 | 0.21 | **0.44** | 0.15 | 0.05 |
| Alcohol/drugs | 7 | 0.09 | 0.19 | 0.11 | 0.17 | **0.41** | 0.09 |
|  | 21 | -0.02 | 0.02 | 0.07 | 0.09 | **0.38** | 0.03 |
|  | 31 | -0.07 | 0.08 | 0.03 | 0.15 | **0.41** | -0.04 |
| Victim understanding | 11 | 0.15 | 0.06 | 0.10 | 0.002 | 0.06 | **0.32** |
|  | 15 | 0.17 | 0.09 | 0.06 | 0.13 | -0.07 | **0.34** |
|  | 16 | 0.13 | 0.13 | 0.09 | 0.009 | 0.08 | **0.40** |

Correlations of items with their parent subscale (corrected for overlap) are in bold.
