# Supplementary material for: Measuring the readiness to screen and manage intimate partner violence: Cross-cultural adaptation and psychometric evaluation of the PREMIS tool for perinatal care providers
Source: PLoS One. 2021 Nov 4;16(11):e0258943. doi: 10.1371/journal.pone.0258943 (PMC8568123; doi:10.1371/journal.pone.0258943)
Supplement: S1 File — (DOCX) [file pone.0258943.s003.docx]

**PREPARATION DES PROFESSIONELS DE SANTE CONCERNANT**

**LA PRISE EN CHARGE DE LA VIOLENCE CONJUGALE**

Une réponse spontanée de votre part au questionnaire suivant nous sera d’une grande aide pour améliorer l’identification et la prise en charge par les professionnels de santé des blessures et des maladies liées à la violence conjugale ^[[1]](#footnote-1)^. Donnez la réponse qui vous vient spontanément même si vous pensez qu'elle n'est pas « politiquement correcte ». N’essayez pas d’imaginer ce que « pourrait » être la bonne réponse attendue.

Ce questionnaire est entièrement anonyme, toutes les réponses resteront confidentielles.

Certaines questions peuvent vous paraître redondantes, toutefois nous vous demandons de répondre à chacune d’entre elles afin d’améliorer la validité du questionnaire. Nous vous remercions de prendre le temps (environ 25 minutes) de répondre à ce questionnaire.

**Partie I : Votre Profil**

**1.** Votre âge: ans

**2.** Genre : 🞏 Homme 🞏 Femme

**3.** Département où vous exercez :

**4.** Quelle est votre secteur d’activité professionnelle ?

🞏 Médecine interne 🞏 Médecine d'urgence

🞏 Médecine générale 🞏 Chirurgie

🞏 Pédiatrie 🞏 Gynécologie / Obstétrique / Maïeutique

🞏 Psychiatrie 🞏 Autre (*précisez* : __________________________________ )

**5.** En quelle année avez-vous obtenu votre diplôme d’exercice professionnel ?

**6.** Combien de patients prenez-vous en charge en moyenne par semaine ? (*une seule réponse possible*)

🞏 Je ne vois pas de patient 🞏 40 – 59

🞏 Moins de 20 🞏 60 ou plus

🞏 20 – 39

**7.** Vous y compris, combien y a-t-il de professionnels de santé sur votre lieu de travail ?

- 1. Médecins :
  2. Sages-femmes :
  3. Infirmiers(ères) (spécialisés ou non) ?

**8.** Vous y compris, combien de professionnels de santé sur votre lieu de travail ont déjà suivi une formation sur la violence conjugale au cours des 6 derniers mois ?

Ceci représente : 🞏 Tous les professionnels 🞏 La plupart d'entre eux

🞏 Certains d'entre eux 🞏 Quelques-uns seulement 🞏 Je ne sais pas

**Partie II : Expérience**

1. Quelle(s) formation(s) en lien avec les violences conjugales avez-vous déjà reçu ? *(plusieurs réponses possibles)*

🞏 Aucune 🞏 J'ai lu le protocole de mon institution

🞏 J'ai visionné une vidéo sur le sujet 🞏 J’ai assisté à une conférence ou à un exposé

🞏 J’ai assisté à un atelier ou à un séminaire 🞏 Formation au cours de mes études

🞏 Formation au cours de mon internat/résidanat/post-internat

🞏 Formation continue 🞏 Autre formation approfondie (plus de 4h)

🞏 Autre (*précisez :* ____________________________________________________________ )

1. Nombre total d'heures de formation estimé sur la violence conjugale : heures
2. Veuillez cocher la case qui décrit le mieux la manière dont vous vous sentez préparé(e) aux tâches suivantes :

|  |  | **Pas préparé(e)** | **Très peu préparé(e)** | **Peu préparé(e)** | **Moyennement préparé(e)** | **Plutôt bien préparé(e)** | **Bien préparé(e)** | **Tout à fait bien préparé(e)** |
| --- | --- | --- | --- | --- | --- | --- | --- | --- |
| **a.** | Poser des questions appropriées concernant la violence conjugale | 🞏 | 🞏 | 🞏 | 🞏 | 🞏 | 🞏 | 🞏 |
| **b.** | Répondre de manière appropriée aux divulgations de maltraitance | 🞏 | 🞏 | 🞏 | 🞏 | 🞏 | 🞏 | 🞏 |
| **c.** | Identifier des signes de violence conjugale en se basant sur les antécédents du patient et l’examen clinique | 🞏 | 🞏 | 🞏 | 🞏 | 🞏 | 🞏 | 🞏 |
| **d.** | Évaluer la volonté d'une victime de violence conjugale à changer sa situation | 🞏 | 🞏 | 🞏 | 🞏 | 🞏 | 🞏 | 🞏 |
| **e.** | Aider une victime de violence conjugale à évaluer son propre danger de mort | 🞏 | 🞏 | 🞏 | 🞏 | 🞏 | 🞏 | 🞏 |
| **f.** | Évaluer la sécurité des enfants de la victime | 🞏 | 🞏 | 🞏 | 🞏 | 🞏 | 🞏 | 🞏 |
| **g.** | Aider une victime de violence conjugale à élaborer un plan de mise en sécurité | 🞏 | 🞏 | 🞏 | 🞏 | 🞏 | 🞏 | 🞏 |
| **h.** | Consigner les antécédents de violence conjugale et les informations relatives à l’examen clinique dans le dossier du patient | 🞏 | 🞏 | 🞏 | 🞏 | 🞏 | 🞏 | 🞏 |
| **i.** | Orienter la victime vers les services appropriés en charge de la violence conjugale | 🞏 | 🞏 | 🞏 | 🞏 | 🞏 | 🞏 | 🞏 |
| **j.** | Répondre aux exigences légales de signalement pour : |  |  |  |  |  |  |  |
|  | - la violence conjugale | 🞏 | 🞏 | 🞏 | 🞏 | 🞏 | 🞏 | 🞏 |
|  | - la maltraitance des personnes âgées | 🞏 | 🞏 | 🞏 | 🞏 | 🞏 | 🞏 | 🞏 |
|  | - la maltraitance des mineurs | 🞏 | 🞏 | 🞏 | 🞏 | 🞏 | 🞏 | 🞏 |

1. Selon vous, quel est votre degré de connaissance concernant les points suivants :

|  |  | **Nul** | **Très limité** | **Limité** | **Moyen** | **Plutôt élevé** | **Elevé** | **Très élevé** |
| --- | --- | --- | --- | --- | --- | --- | --- | --- |
| **a.** | Les exigences légales de signalement en matière de : |  |  |  |  |  |  |  |
|  | - violence conjugale | 🞏 | 🞏 | 🞏 | 🞏 | 🞏 | 🞏 | 🞏 |
|  | - maltraitance des mineurs | 🞏 | 🞏 | 🞏 | 🞏 | 🞏 | 🞏 | 🞏 |
|  | - maltraitance des personnes âgées | 🞏 | 🞏 | 🞏 | 🞏 | 🞏 | 🞏 | 🞏 |
| **b.** | Les signes ou symptômes de violence conjugale | 🞏 | 🞏 | 🞏 | 🞏 | 🞏 | 🞏 | 🞏 |
| **c.** | Comment documenter un cas de violence conjugale dans le dossier d'un patient | 🞏 | 🞏 | 🞏 | 🞏 | 🞏 | 🞏 | 🞏 |
| **d.** | Les services appropriés prenant en charge les victimes de violence conjugale | 🞏 | 🞏 | 🞏 | 🞏 | 🞏 | 🞏 | 🞏 |
| **e.** | Les auteurs de violence conjugale | 🞏 | 🞏 | 🞏 | 🞏 | 🞏 | 🞏 | 🞏 |
| **f.** | Les liens entre violence conjugale et grossesse | 🞏 | 🞏 | 🞏 | 🞏 | 🞏 | 🞏 | 🞏 |
| **g.** | Reconnaître les conséquences, pour un enfant, d’être témoin de violence conjugale | 🞏 | 🞏 | 🞏 | 🞏 | 🞏 | 🞏 | 🞏 |
| **h.** | Les questions à poser pour identifier les violences conjugales | 🞏 | 🞏 | 🞏 | 🞏 | 🞏 | 🞏 | 🞏 |
| **i.** | Les raisons pour lesquelles une victime de violence conjugale choisit de ne pas en parler | 🞏 | 🞏 | 🞏 | 🞏 | 🞏 | 🞏 | 🞏 |
| **j.** | Votre rôle dans la détection des cas de violence conjugale | 🞏 | 🞏 | 🞏 | 🞏 | 🞏 | 🞏 | 🞏 |
| **k.** | Que dire et ne pas dire face à un patient victime de violence conjugale | 🞏 | 🞏 | 🞏 | 🞏 | 🞏 | 🞏 | 🞏 |
| **l.** | Identifier les dangers pour un patient victime de violence conjugale | 🞏 | 🞏 | 🞏 | 🞏 | 🞏 | 🞏 | 🞏 |
| **m.** | Elaborer un plan de mise en sécurité avec une victime de violence conjugale | 🞏 | 🞏 | 🞏 | 🞏 | 🞏 | 🞏 | 🞏 |
| **n.** | La compréhension que la victime de violence conjugale a de sa situation ; les étapes par lesquelles elle peut passer et sa volonté de changer sa situation | 🞏 | 🞏 | 🞏 | 🞏 | 🞏 | 🞏 | 🞏 |

**Partie III : Connaissances sur la violence conjugale**

1. Parmi les facteurs de risque pour une personne de devenir victime de violence conjugale, lequel est le plus déterminant ? (*une seule réponse possible)*

🞏 Âge (<30 ans) 🞏 Toxicomanie/alcoolisme du conjoint

🞏 Etre une femme 🞏 Antécédents familiaux de maltraitance

🞏 Je ne sais pas

1. Laquelle de ces propositions est généralement vraie au sujet des auteurs de violences ? (*une seule réponse possible*)

🞏 Ils ont du mal à maîtriser leur colère

🞏 Ils utilisent la violence comme un moyen de contrôle sur leur conjoint

🞏 Ils sont violents car ils consomment de l'alcool ou des drogues

🞏 Ils entrent en conflit avec n’importe qui

1. Parmi les propositions suivantes, lesquelles sont un signe d’alerte qu'un patient peut avoir subi des violences de la part de son conjoint ? *(plusieurs réponses possibles)*

🞏 Douleurs chroniques inexpliquées 🞏 Anxiété 🞏 Abus de substances

🞏 Blessures fréquentes 🞏 Dépression

1. Parmi les propositions suivantes, quels motifs peuvent empêcher une victime de violence conjugale de se sortir d’une relation violente ? *(plusieurs réponses possibles*)

🞏 Peur des représailles 🞏 Dépendance financière vis-à-vis de l'auteur de violence

🞏 Croyances religieuses 🞏 Besoins des enfants

🞏 Amour pour son conjoint 🞏 Isolement

1. Parmi les questions suivantes, lesquelles sont les plus appropriées pour aborder le sujet des violences conjugales ? *(plusieurs réponses possibles)*

🞏 « Êtes-vous victime de violence conjugale ? »

🞏 « Votre conjoint(e) vous a-t-il déjà blessé(e) ou menacé(e) ? »

🞏 « Avez-vous déjà eu peur de votre conjoint(e)? »

🞏 « Votre conjoint(e) vous a-t-il déjà frappé(e) ou blessé(e) ? »

1. Parmi les propositions suivantes, lesquelles sont généralement vraies ? *(plusieurs réponses possibles)*

🞏 Il existe des cas fréquents de patients victimes de maltraitance ne présentant pas de blessures apparentes

🞏 Il existe des schémas comportementaux au sein d'un couple qui peuvent indiquer une situation de violence conjugale

🞏 Des zones spécifiques du corps sont plus souvent visées dans les cas de violence conjugale

🞏 Certains types de blessures sont fréquemment liés à la violence conjugale

🞏 Des blessures à différents stades de guérison peuvent être un signe de maltraitance

1. Veuillez associer les comportements et sentiments suivants des patients ayant des antécédents de violence conjugale, avec le stade d'évolution correspondant :

| 1 *= Déni* | 2 *= Réflexion* | 3 *= Préparation* |
| --- | --- | --- |
| 4 *= Action* | 5 *= Poursuite de l’action* | 6 *= Fin* |

Commence à envisager à un moyen de quitter le(la) conjoint(e) violent(e)

Nie qu'il y a un problème

Commence à penser que ce n'est pas de sa faute si il/elle est victime de maltraitance

Continue à changer ses comportements

Obtient une ordonnance de protection

1. Veuillez cocher la case qui correspond le mieux à votre réponse:

|  |  | **Vrai** | **Faux** | **Je ne sais pas** |
| --- | --- | --- | --- | --- |
| **a.** | La consommation d'alcool est le meilleur indice de probable présence de violence conjugale | 🞏 | 🞏 | 🞏 |
| **b.** | Il existe de bonnes raisons pour ne pas mettre fin à une relation violente | 🞏 | 🞏 | 🞏 |
| **c.** | Les motifs de suspicion de violence conjugale ne doivent pas figurer dans le dossier du patient si celui-ci n'en parle pas | 🞏 | 🞏 | 🞏 |
| **d.** | Lorsque les professionnels de santé interrogent les patients sur les violences conjugales, ils doivent utiliser les termes « maltraité(e) » ou « battu(e) » | 🞏 | 🞏 | 🞏 |
| **e.** | Soutenir un patient dans son choix de rester dans une relation violente revient à fermer les yeux sur la maltraitance | 🞏 | 🞏 | 🞏 |
| **f.** | Les victimes de violence conjugale sont capables de faire des choix appropriés quant à la façon de gérer leur situation | 🞏 | 🞏 | 🞏 |
| **g.** | Les professionnels de santé ne doivent pas forcer les patients à reconnaître qu’ils vivent une relation de maltraitance | 🞏 | 🞏 | 🞏 |
| **h.** | Les victimes de violence conjugale courent un risque plus important de blessure lorsqu'elles quittent leur conjoint | 🞏 | 🞏 | 🞏 |
| **i.** | Les blessures par strangulation sont rares dans les cas de violence conjugale | 🞏 | 🞏 | 🞏 |
| **j.** | Autoriser la présence du conjoint ou d'amis pendant la consultation assure la sécurité d’une victime de violence conjugale | 🞏 | 🞏 | 🞏 |
| **k.** | Si l'enfant ne court pas un danger immédiat, les professionnels de santé ne doivent pas effectuer un signalement de cas d’enfants témoin de violence conjugale auprès de la Cellule de Recueil des Informations Préoccupantes (CRIP) | 🞏 | 🞏 | 🞏 |

**Partie IV: Opinions**

Pour chacune des propositions suivantes, veuillez cocher la case qui correspond le mieux à votre réponse:

|  |  | **Pas du tout d’accord** | **Pas d’accord** | **Plutôt pas d’accord** | **Ni pas d’accord, ni d’accord** | **Plutôt d’accord** | **D’accord** | **Tout à fait d’accord** |
| --- | --- | --- | --- | --- | --- | --- | --- | --- |
| **1.** | Si une victime de violence conjugale ne reconnait pas être victime de maltraitance, il n'y a pas grand-chose que je puisse faire pour l'aider | 🞏 | 🞏 | 🞏 | 🞏 | 🞏 | 🞏 | 🞏 |
| **2.** | Je demande à tous mes nouveaux patients si de la maltraitance existe au sein du couple | 🞏 | 🞏 | 🞏 | 🞏 | 🞏 | 🞏 | 🞏 |
| **3.** | Sur mon lieu de travail, on m'encourage à agir en cas de violence conjugale | 🞏 | 🞏 | 🞏 | 🞏 | 🞏 | 🞏 | 🞏 |
| **4.** | Je peux orienter une victime de violence conjugale vers les services locaux appropriés en charge de la violence conjugale | 🞏 | 🞏 | 🞏 | 🞏 | 🞏 | 🞏 | 🞏 |
| **5.** | Je suis capable d'identifier un cas de violence conjugale sans interroger directement mon patient | 🞏 | 🞏 | 🞏 | 🞏 | 🞏 | 🞏 | 🞏 |
| **6.** | Je ne suis pas suffisamment formé(e) pour aider une personne à aborder une situation de violence conjugale | 🞏 | 🞏 | 🞏 | 🞏 | 🞏 | 🞏 | 🞏 |
| **7.** | Les patients alcooliques ou toxicomanes sont susceptibles d’avoir des antécédents de violence conjugale | 🞏 | 🞏 | 🞏 | 🞏 | 🞏 | 🞏 | 🞏 |
| **8.** | Les victimes de violence conjugale ont le droit de prendre leurs propres décisions quant à l'intervention ou non de l’équipe soignante | 🞏 | 🞏 | 🞏 | 🞏 | 🞏 | 🞏 | 🞏 |
| **9.** | Je me sens à l'aise pour parler de violence conjugale avec mes patients | 🞏 | 🞏 | 🞏 | 🞏 | 🞏 | 🞏 | 🞏 |
| **10.** | Je n'ai pas les compétences nécessaires pour parler de maltraitance avec une victime de violence conjugale qui est : |  |  |  |  |  |  |  |
|  | a) une femme | 🞏 | 🞏 | 🞏 | 🞏 | 🞏 | 🞏 | 🞏 |
|  | b) un homme | 🞏 | 🞏 | 🞏 | 🞏 | 🞏 | 🞏 | 🞏 |
|  | c) de culture/d'ethnie différente | 🞏 | 🞏 | 🞏 | 🞏 | 🞏 | 🞏 | 🞏 |
| **11.** | Si les victimes de maltraitance décident de poursuivre leur relation après des épisodes répétés de violence, elles doivent accepter la responsabilité de cette violence | 🞏 | 🞏 | 🞏 | 🞏 | 🞏 | 🞏 | 🞏 |

|  |  | **Pas du tout d’accord** | **Pas d’accord** | **Plutôt pas d’accord** | **Ni pas d’accord, ni d’accord** | **Plutôt d’accord** | **D’accord** | **Tout à fait d’accord** |
| --- | --- | --- | --- | --- | --- | --- | --- | --- |
| **12.** | Je connais les obligations légales en cas de suspicion de |  |  |  |  |  |  |  |
|  | a) violence conjugale | 🞏 | 🞏 | 🞏 | 🞏 | 🞏 | 🞏 | 🞏 |
|  | b) maltraitance sur mineurs | 🞏 | 🞏 | 🞏 | 🞏 | 🞏 | 🞏 | 🞏 |
|  | c) maltraitance sur personnes âgées | 🞏 | 🞏 | 🞏 | 🞏 | 🞏 | 🞏 | 🞏 |
| **13.** | Les professionnels de santé n'ont pas le temps d'aider les patients à aborder une situation de violence conjugale | 🞏 | 🞏 | 🞏 | 🞏 | 🞏 | 🞏 | 🞏 |
| **14.** | Je suis capable d'obtenir les informations nécessaires pour identifier la violence conjugale comme cause sous-jacente de maladies du patient (par exemple, dépression, migraines) | 🞏 | 🞏 | 🞏 | 🞏 | 🞏 | 🞏 | 🞏 |
| **15.** | Si un patient refuse de parler de la maltraitance, les professionnels de santé ne peuvent traiter que ses blessures | 🞏 | 🞏 | 🞏 | 🞏 | 🞏 | 🞏 | 🞏 |
| **16.** | Les victimes de maltraitance pourraient mettre un terme à leur relation de couple si elles le voulaient | 🞏 | 🞏 | 🞏 | 🞏 | 🞏 | 🞏 | 🞏 |
| **17.** | Je me conforme aux exigences de signalement de cas de violence conjugale en accord avec la loi | 🞏 | 🞏 | 🞏 | 🞏 | 🞏 | 🞏 | 🞏 |
| **18.** | Les professionnels de santé ont la responsabilité d’interroger tous les patients sur la violence conjugale | 🞏 | 🞏 | 🞏 | 🞏 | 🞏 | 🞏 | 🞏 |
| **19.** | Mon organisation professionnelle me permet d’avoir suffisamment de temps pour prendre en charge des victimes de violence conjugale | 🞏 | 🞏 | 🞏 | 🞏 | 🞏 | 🞏 | 🞏 |
| **20.** | J'ai contacté les services locaux appropriés en charge de la violence conjugale afin de leur adresser une victime de violence conjugale | 🞏 | 🞏 | 🞏 | 🞏 | 🞏 | 🞏 | 🞏 |
| **21.** | L'abus d'alcool est une cause majeure de violence conjugale. | 🞏 | 🞏 | 🞏 | 🞏 | 🞏 | 🞏 | 🞏 |
| **22.** | Les victimes de maltraitance ont souvent de bonnes raisons de rester dans la relation de maltraitance | 🞏 | 🞏 | 🞏 | 🞏 | 🞏 | 🞏 | 🞏 |
| **23.** | Je suis trop occupé(e) pour faire partie d'une équipe multidisciplinaire qui prend en charge des cas de violence conjugale | 🞏 | 🞏 | 🞏 | 🞏 | 🞏 | 🞏 | 🞏 |
| **24.** | Le dépistage de la violence conjugale est susceptible d’offenser ceux qui en font l’objet | 🞏 | 🞏 | 🞏 | 🞏 | 🞏 | 🞏 | 🞏 |
| **25.** | Je dispose d'un espace privé adéquat pour prendre en charge les victimes de violence conjugale | 🞏 | 🞏 | 🞏 | 🞏 | 🞏 | 🞏 | 🞏 |

|  |  | **Pas du tout d’accord** | **Pas d’accord** | **Plutôt pas d’accord** | **Ni pas d’accord, ni d’accord** | **Plutôt d’accord** | **D’accord** | **Tout à fait d’accord** |
| --- | --- | --- | --- | --- | --- | --- | --- | --- |
| **26.** | Je suis capable d'obtenir les informations nécessaires pour identifier la violence conjugale comme cause sous-jacente de blessures du patient (par exemple, hématomes, fractures, etc.) | 🞏 | 🞏 | 🞏 | 🞏 | 🞏 | 🞏 | 🞏 |
| **27.** | Une femme qui choisit de s’écarter des rôles traditionnels est davantage concernée par la violence conjugale | 🞏 | 🞏 | 🞏 | 🞏 | 🞏 | 🞏 | 🞏 |
| **28.** | Les professionnels de santé n’ont pas les connaissances nécessaires pour aider les patients à aborder une situation de violence conjugale | 🞏 | 🞏 | 🞏 | 🞏 | 🞏 | 🞏 | 🞏 |
| **29.** | Je peux adapter mon intervention en fonction de la volonté d'une victime de violence conjugale à changer sa situation | 🞏 | 🞏 | 🞏 | 🞏 | 🞏 | 🞏 | 🞏 |
| **30.** | Je comprends pourquoi les victimes de violence conjugale ne suivent pas toujours les recommandations de l’équipe soignante | 🞏 | 🞏 | 🞏 | 🞏 | 🞏 | 🞏 | 🞏 |
| **31.** | La consommation d’alcool ou autre drogue est liée au fait d’être victime de violence conjugale | 🞏 | 🞏 | 🞏 | 🞏 | 🞏 | 🞏 | 🞏 |
| **32.** | Je peux reconnaître des victimes de violence conjugale à leur manière de se comporter | 🞏 | 🞏 | 🞏 | 🞏 | 🞏 | 🞏 | 🞏 |

**Partie V : Questions liées à votre exercice**

1. Au cours des 6 derniers mois, combien de *nouveaux diagnostics* de violence conjugale (identification d’un cas grave, découverte d’abus actuels, ou révélation par le patient d’antécédents) estimez-vous avoir fait ?

🞏 Aucun 🞏 11 – 20

🞏 1 – 5 🞏 21 ou plus

🞏 6 – 10 🞏 Non applicable - pas de pratique clinique

1. Cochez les situations listées ci-dessous pour lesquelles vous dépistez la violence conjugale (*plusieurs réponses possibles*)

🞏 Non applicable - pas de pratique clinique

🞏 Je ne fais pas de dépistage à l’heure actuelle

🞏 Je dépiste tous les nouveaux patients

🞏 Je dépiste tous les patients qui sont des femmes

🞏 Je dépiste tous les patients avec des signes de maltraitance dans les antécédents médicaux ou à l’examen clinique

🞏 Je dépiste tous les patients qui sont des femmes au moment de leur examen annuel

🞏 Je dépiste toutes les femmes enceintes à des moments clés de leur grossesse

🞏 Je dépiste régulièrement tous les patients

🞏 Je dépiste certaines catégories de patients uniquement (*cochez ci-dessous*) :

🞏 Adolescent(e)s

🞏 Jeunes femmes majeures (moins de 30 ans)

🞏 Femmes âgées de plus de 65 ans

🞏 Femmes célibataires ou divorcées

🞏 Femmes mariées

🞏 Femmes alcooliques ou toxicomanes

🞏 Mères célibataires

🞏 Femmes immigrées

🞏 Femmes homosexuelles

🞏 Hommes homosexuels

🞏 Femmes déprimées/suicidaires

🞏 Femmes enceintes

🞏 Mères de tous mes patients mineurs (le cas échéant)

🞏 Mères de tous mes patients mineurs montrant des signes indiquant qu'ils ont été témoins de violence conjugale

🞏 Mères d’enfants victimes de maltraitance ou de négligence suspectée ou confirmée

🞏 Autre - *Veuillez préciser* : _____________________________________________

_____________________________________________

_____________________________________________

_____________________________________________

1. Au cours des 6 derniers mois, parmi les patients présentant les signes suivants, à quelle fréquence les avez-vous interrogés sur de possibles violences conjugales :

|  |  | **Jamais** | **Rarement** | **Parfois** | **Presque toujours** | **Toujours** | **Non applicable** |
| --- | --- | --- | --- | --- | --- | --- | --- |
| **a.** | Des blessures | 🞏 | 🞏 | 🞏 | 🞏 | 🞏 | 🞏 |
| **b.** | Une douleur pelvienne chronique | 🞏 | 🞏 | 🞏 | 🞏 | 🞏 | 🞏 |
| **c.** | Un syndrome du côlon irritable | 🞏 | 🞏 | 🞏 | 🞏 | 🞏 | 🞏 |
| **d.** | Des céphalées | 🞏 | 🞏 | 🞏 | 🞏 | 🞏 | 🞏 |
| **e.** | Une dépression/anxiété | 🞏 | 🞏 | 🞏 | 🞏 | 🞏 | 🞏 |
| **f.** | Une hypertension | 🞏 | 🞏 | 🞏 | 🞏 | 🞏 | 🞏 |
| **g.** | Des troubles alimentaires | 🞏 | 🞏 | 🞏 | 🞏 | 🞏 | 🞏 |

1. Au cours des 6 derniers mois, quelle(s) mesure(s) avez-vous prise(s) après avoir identifié un cas de violence conjugale ? (*plusieurs réponses possibles*)

🞏 Je n’ai pas identifié de violence conjugale au cours des 6 derniers mois

🞏 J'ai fourni des informations (numéros de téléphone, brochures, autres informations) au patient

🞏 J'ai conseillé le patient sur les différentes options qu’il/elle avait

🞏 J'ai évalué la sécurité de la victime

🞏 J'ai évalué la sécurité des enfants de la victime

🞏 J'ai aidé le patient à élaborer un plan personnel de mise en sécurité

🞏 J'ai orienté le patient vers :

🞏 Une thérapie individuelle 🞏 Un juriste/un avocat

🞏 Une thérapie de couple 🞏 Une thérapie pour enfants/groupe de parole

🞏 Un assistant social/juriste local 🞏 Un responsable/organisme religieux

🞏 Un addictologue 🞏 La police ou gendarmerie

🞏 Un programme de prise en charge des auteurs de violence

🞏 Un centre d'accueil pour femmes battues

🞏 Un groupe de soutien pour femmes battues

🞏 La hotline nationale pour les victimes de violence conjugale

🞏 Un groupe de soutien pour homosexuel(le)s, transsexuels, bisexuels

🞏 Un organisme d’aide (hébergement, éducation, emploi, financier…)

🞏 Autre orientation (*précisez* : ____________________________________________

____________________________________________

____________________________________________ )

🞏 Autre action (*précisez* : _______________________________________________

_______________________________________________

_______________________________________________ )

1. Existe-t-il un protocole de prise en charge des victimes de violence conjugale majeures au sein de votre lieu de travail ? (*une seule réponse possible*)

🞏 Oui, et il est largement utilisé 🞏 Non

🞏 Oui, et il est utilisé dans une certaine mesure 🞏 Je ne suis pas sûr(e)

🞏 Oui, mais il n'est pas utilisé 🞏 Ne concerne pas ma patientèle

🞏 Non applicable - pas de pratique clinique actuellement

1. Etes-vous familier des politiques de dépistage et de prise en charge des victimes de violence conjugale au sein de l’établissement dans lequel vous exercez ?

🞏 Oui 🞏 Non 🞏 Non applicable

1. Y a t-il un appareil photo sur votre lieu de travail pour prendre en photo les blessures des victimes de violence conjugale ?

🞏 Oui 🞏 Je ne suis pas sûr(e)

🞏 Non 🞏 Ne concerne pas ma patientèle

🞏 Non applicable - pas de pratique clinique actuellement

1. Pour chaque victime de violence conjugale que vous avez identifiée au cours des 6 derniers mois, à quelle fréquence avez-vous :

|  |  | **Jamais** | **Rarement** | **Parfois** | **Presque toujours** | **Toujours** | **Non applicable** |
| --- | --- | --- | --- | --- | --- | --- | --- |
| **a.** | Consigné les déclarations du patient concernant les violences conjugales dans son dossier médical | 🞏 | 🞏 | 🞏 | 🞏 | 🞏 | 🞏 |
| **b.** | Utilisé un schéma corporel pour documenter les blessures d’un patient | 🞏 | 🞏 | 🞏 | 🞏 | 🞏 | 🞏 |
| **c.** | Photographié les blessures d’une victime pour les inclure dans son dossier médical | 🞏 | 🞏 | 🞏 | 🞏 | 🞏 | 🞏 |
| **d.** | Effectué un signalement aux autorités compétentes en cas d’obligation légale | 🞏 | 🞏 | 🞏 | 🞏 | 🞏 | 🞏 |
| **e.** | Evalué la sécurité de la victime | 🞏 | 🞏 | 🞏 | 🞏 | 🞏 | 🞏 |
| **f.** | Evalué la sécurité des enfants de la victime | 🞏 | 🞏 | 🞏 | 🞏 | 🞏 | 🞏 |
| **g.** | Aidé une victime de violence conjugale à élaborer un plan de mise en sécurité | 🞏 | 🞏 | 🞏 | 🞏 | 🞏 | 🞏 |
| **h.** | Contacté un organisme spécialisé dans la prise en charge des violences conjugales | 🞏 | 🞏 | 🞏 | 🞏 | 🞏 | 🞏 |
| **i.** | Fait preuve de compréhension et de soutien dans votre discours | 🞏 | 🞏 | 🞏 | 🞏 | 🞏 | 🞏 |
| **j.** | Fourni des informations de base sur la violence conjugale | 🞏 | 🞏 | 🞏 | 🞏 | 🞏 | 🞏 |
| **k.** | Adressé le patient à un spécialiste et/ou donné d’autres sources d’informations | 🞏 | 🞏 | 🞏 | 🞏 | 🞏 | 🞏 |

1. Y a-t-il de la documentation (affiches, brochures, etc.) à la disposition des patients victimes de violence conjugale sur votre lieu de travail ? (*une seule réponse possible*)

🞏 Oui, bien en vue et utilisé par les patients

🞏 Oui, bien en vue et mais non utilisé par les patients

🞏 Oui, mais non mise en évidence

🞏 Non

🞏 Je ne suis pas sûr(e)

🞏 Ne concerne pas ma patientèle

🞏 Non applicable - pas de pratique clinique actuellement

1. Donnez-vous de la documentation sur les violences conjugales (affiches, brochures, etc.) aux patients victimes ? (*une seule réponse possible*)

🞏 Oui, presque toujours

🞏 Oui, lorsque cela est sans risque pour le patient mais non accessible directement aux patients

🞏 Oui, mais seulement à la demande du patient

🞏 Non, en raison de l'absence de ressources locales adéquates

🞏 Non, parce que je ne trouve pas que cette documentation soit utile en général

🞏 Non, pour d’autres raisons (*précisez* : ___________________________________________

___________________________________________

___________________________________________ )

🞏 Ne concerne pas ma patientèle

🞏 Non applicable - pas de pratique clinique actuellement

1. Considérez-vous que vous disposez de spécialistes adéquats auxquels adresser vos patients majeurs victimes de violence conjugale **sur votre lieu de travail** (y compris psychiatres) ?

🞏 Oui

🞏 Non

🞏 Je ne suis pas sûr(e)

🞏 Non applicable - pas de pratique clinique actuellement

🞏 Ne concerne pas ma patientèle

1. Considérez-vous que vous connaissez des recours adéquats auxquels adresser vos patients majeurs victimes de violence conjugale **dans votre région** (y compris centre d’accueil ou association) ?

🞏 Oui

🞏 Non

🞏 Je ne suis pas sûr(e)

🞏 Non applicable - pas de pratique clinique actuellement

🞏 Ne concerne pas ma patientèle

**Nous vous remercions vivement d’avoir répondu à ce questionnaire.**

1. La violence conjugale (aussi communément appelée violence domestique, violence exercée sur le conjoint ou violence familiale) correspond à la violence exercée au sein d'un couple, marié/pacsé ou non. [↑](#footnote-ref-1)
