# Supplementary material for: Measuring the readiness to screen and manage intimate partner violence: Cross-cultural adaptation and psychometric evaluation of the PREMIS tool for perinatal care providers
Source: PLoS One. 2021 Nov 4;16(11):e0258943. doi: 10.1371/journal.pone.0258943 (PMC8568123; doi:10.1371/journal.pone.0258943)
Supplement: Attachment — Submitted filename: Response to Reviewers.docx [file pone.0258943.s005.docx]

Mrs. Candy Guiguet-Auclair

CHU de Clermont-Ferrand

7 place Henri Dunant

63000 Clermont-Ferrand

France

To Stefano Federici, Academic Editor of Plos One

October, 08^th^ 2021

Dear Academic Editor,

We thank the reviewer for his thorough comments on the manuscript "Measuring the readiness to screen and manage intimate partner violence: cross-cultural adaptation and psychometric evaluation of the PREMIS tool for perinatal care providers" [Ref.: PONE-D-21-19470R1].

We carefully revise our manuscript to respond to the reviewer’s comments and journal requirements. Modifications to the manuscript are highlighted in yellow in the revised manuscript with track changes.

Sincerely yours.

Mrs. Candy Guiguet-Auclair

**Journal Requirements**

Please review your reference list to ensure that it is complete and correct. If you have cited papers that have been retracted, please include the rationale for doing so in the manuscript text, or remove these references and replace them with relevant current references. Any changes to the reference list should be mentioned in the rebuttal letter that accompanies your revised manuscript. If you need to cite a retracted article, indicate the article’s retracted status in the References list and also include a citation and full reference for the retraction notice.

Response: We have reviewed all the references in the manuscript and we have modified the formatting of some of them, in order to be conform with the “Vancouver” style.

**Reviewer' comments**

Regarding the abstract, I would substitute the following sentence "Results: The PREMIS was successfully translated and cross-culturally adapted into French." by "Results: The PREMIS was successfully translated and cross-culturally adapted to the context of metropolitan France.". Indeed, French is spoken in many countries but in different contexts.

Response: We modified the first sentence of the Results section in the abstract as recommended.
